# Is infant neural sensitivity to vocal emotion associated with mother-infant relational experience?

**DOI:** 10.1371/journal.pone.0212205

**Published:** 2019-02-27

**Authors:** Chen Zhao, Georgia Chronaki, Ingo Schiessl, Ming Wai Wan, Kathryn M. Abel

**Affiliations:** 1 Centre for Women’s Mental Health, Faculty of Biology, Medicine and Health, University of Manchester, Manchester, United Kingdom; 2 Developmental Cognitive Neuroscience (DCN) Laboratory, School of Psychology, University of Central Lancashire, Preston, United Kingdom; 3 Division of Neuroscience & Experimental Psychology, Faculty of Biology, Medicine and Health, University of Manchester, Manchester, United Kingdom; 4 Developmental Brain-Behaviour Laboratory, Psychology, University of Southampton, United Kingdom; 5 Greater Manchester Mental Health NHS Foundation Trust, Manchester, United Kingdom; Victoria University of Wellington, NEW ZEALAND

## Abstract

An early understanding of others’ vocal emotions provides infants with a distinct advantage for eliciting appropriate care from caregivers and for navigating their social world. Consistent with this notion, an emerging literature suggests that a temporal cortical response to the prosody of emotional speech is observable in the first year of life. Furthermore, neural specialisation to vocal emotion in infancy may vary according to early experience. Neural sensitivity to emotional non-speech vocalisations was investigated in 29 six-month-old infants using near-infrared spectroscopy (fNIRS). Both angry and happy vocalisations evoked increased activation in the temporal cortices (relative to neutral and angry vocalisations respectively), and the strength of the angry minus neutral effect was positively associated with the degree of directiveness in the mothers’ play interactions with their infant. This first fNIRS study of infant vocal emotion processing implicates bilateral temporal mechanisms similar to those found in adults and suggests that infants who experience more directive caregiving or social play may more strongly or preferentially process vocal anger by six months of age.

## Introduction

Human responsiveness to familiar vocalisations starts prenatally when the heart rate of the fetus increases in response to the mother’s voice compared to that of an unknown female [1]. The ability to discriminate vocal emotion as early as possible in life serves an adaptive evolutionary function [2]. Infants rely heavily on their mothers’ emotional prosody, such as affective warmth or fear, as a basis to elicit care and, ultimately, to maintain safety from threat [3, 4]. Positive vocalisations are likely to facilitate infant-mother bonding and secure attachment [3, 5, 6] and infants will be familiar with their mothers use of infant-directed speech, a style often characterised by exaggerated positive affect [7, 8]. On the other hand, negative vocalisations, especially angry ones, act as a direct cue to react to or avoid dangerous situations [9, 10]. The auditory processing of vocal emotion is likely to be rudimentary in the early months [11]; then at around 5 months, the ability to discriminate vocal affective expressions generalises to non-caregiver female voices [12–16]. Soon after, infants develop the ability to ‘social reference’ known adults to gain vocal and facial information on how to react to ambiguous, potentially threatening situations [17, 18]. Young infants cannot always access others’ facial cues because of their relative immobility, which may partially explain their increased reliance on vocal over facial expression for accurate emotional information [15, 18].

Research on voice processing in the infant brain is relatively new. Evidence from adult neuroimaging implicates a temporo-frontal pathway for the processing of emotional vocalisations: the temporal cortices for the acoustic analysis of vocal stimuli and the frontal regions for more detailed cognitive evaluation (e.g. [19–22]). Informed by adult brain lesion studies, vocal emotion processing was initially thought to be lateralised to the right hemisphere [20–22]. Current evidence supports the crucial role of bilateral superior temporal and inferior frontal regions [23–26], based on paradigms involving varied stimuli (speech and semantic meanings) and task requirements (implicit and explicit tasks).

Consistent with adult findings, functional imaging studies of infant voice processing suggest that the temporal and/or frontal cortical regions are sensitive to voice between ages of 3 and 7 months [27–31]. Two of these studies further report that emotional prosody elicited a stronger response compared to neutral vocalisations in voice-sensitive regions [27, 30]. These findings broadly mirror the timeline suggested by looking-time studies [12–14], and may reveal an early version of the adult temporo-frontal vocal emotion processing pathway [19–22] which prioritises the processing of emotional [26, 32] (especially negative [23, 32, 33]) prosody. In adults, the relatively stronger neural response to vocal negativity likely reflects an attentional bias for negative stimuli [34]. Furthermore, children show this negativity bias in a range of socio-communicative domains, such as social referencing and language acquisition [2, 35]. Studies of infant processing of emotional speech found an increased temporal activation in response to angry and happy speech compared to neutral speech in 7- to 8-month infants [27, 36]. Two functional magnetic resonance imaging (fMRI) studies of non-speech prosody processing in 3- to 7-month sleeping infants reported stronger neural responses to sad than neutral vocalisations [29, 30], this may suggest that infants are able to detect or discriminate emotion within non-speech vocalisations earlier than in speech.

Near Infrared Spectroscopy (NIRS) is a neuroimaging technique that offers distinct advantages for studying infant brain functioning in response to vocal stimuli. Compared with fMRI and elecotroencephalogram (EEG), the equipment is portable, silent (thus ideal for using auditory stimuli), and less intrusive (e.g. the infant can sit on the mother’s lap during measurement); all of which makes fNIRS potentially more suitable for infant studies. However, neither of the infant fNIRS studies on vocal emotion processing to date employed non-speech vocalisations at a time when infants are pre-verbal. One study in sleeping neonates found differentiated neural responses in the temporal cortex to fearful, angry, happy speech compared to neutral speech [37]. A study of 7-month-old awake infants similarly suggest that angry and happy speech evoked stronger frontal and temporal activations compared to neutral speech [27]. The present study sought to extend our current understanding of the emergence of vocal emotion sensitivity by using non-speech stimuli with 6-month-old preverbal infants.

Furthermore, almost no neuroimaging studies have examined whether environmental factors may be associated with individual differences in infant vocal emotion processing. Behavioural studies suggest that both language and relational development are shaped by maternal behaviour toward the infant. For example, qualities of maternal behaviour, such as the degree of behavioural sensitive responding [6, 38, 39], play a significant role in the child’s language development. The precise significance and meaning that infants attach to different vocal emotions may also differ according the qualities of the mother-infant attachment relationship given that infants are highly dependent on maternal communication to maintain safety from threat. From the earliest months of life, infants begin to regulate their own behaviour and emotions according to the quality of care they receive [11]. The emerging ability to process and differentiate vocal emotions may play an important role in communicative and social-emotional development and may be influenced by the affective tendencies of the mother that accompany her caregiving or interactive style. Evidence from EEG studies suggest that maternal caregiving behaviour may relate to longitudinal changes in infants’ frontal resting EEG power, which serves attentional processes [40, 41]. While maternal sensitivity is typically characterised by positive vocal cues from high emotional warmth [3, 42], infants with sensitively responsive mothers may prioritise attention to all strong emotional information as they have learned through experience that others’ vocalisations (and their own) are meaningful and relevant for understanding and navigating their interpersonal relationships and environment.

Another type of caregiving behaviour is described as maternal directiveness, which refers to the amount and severity of vocal or behavioural demands, intrusions or critical utterances used by the mother. Maternal directiveness may be expressed in vocally negative forms and conveys a degree of *expectation* (explicitly or implicitly) that the infant attends to or complies, or prohibits such action [43]. Therefore, exposure to high directiveness over time may plausibly give rise to a bias towards attending to negative prosody that may be observed at a neural level. One study to date has attempted to link maternal behaviour (intrusiveness) with 3- to 7-month-old infant neural vocal response—in infants at high and low risk of autism, and found no significant linear relationship in this specific group [30].

The current study investigated 6-month-old infant hemodynamic response to emotional prosody in non-speech vocalisations. The key objective was to test whether there was increased neural activation in the temporal region in response to emotional (angry, happy) compared to neutral vocalisations, as found in adult studies. Secondly, we explored whether individual variation in neural response to emotional prosody would correlate with infants’ real-life maternal interactions, as measured from independently video-recorded observations of mother-infant play interactions. Specifically, we examined whether the degree of maternal sensitivity and directiveness toward infant was associated with infant neural activation in response to emotional prosody.

## Materials and methods

### Participants

Forty white, fluent English-speaking mothers over 18 years of age were recruited from three community health centres in Manchester, UK. Eligible mothers had no current mental disorder and had given birth to healthy infants. Forty infants (20 boys, and 20 girls) of recruited mothers participated in the current study at 6 months of age. The final sample consisted of 29 infants (see Table 1 for demographics), as 11 infants did not meet the minimum 4 out of 8 trials per experimental condition as a result of motion artefacts. This attrition rate is within the standard range for infant NIRS studies [44]. A power analysis using the G*power program [45] indicated that a sample size of N = 29 would give 92% power to achieve an effect size of 0.59 (which equals to eta-squared of 0.26). All infants were born full term (37–42 weeks gestation) except n = 1 born at 36 weeks gestation (corrected age used), at normal birth weight (>2500g), and had no hearing difficulties according to parent report. The UK National Health Service ethics committee approved the study (ref: 15/NW/0684), and mothers provided consent for their infant’s involvement.

**Table 1:** Sample demographic information (N = 29).

	Mean ± SD	Range
Maternal age (years)	34.79 ± 3.67	23–40
Infant age (days)	189 ± 9.66	175–214
	Demographic category	Count (%)
Infant sex	Female	15 (51.7)
	Male	14 (48.3)
	Category	Frequency
Current maternal work status*	Full-time work	7 (24.14)
	Part-time work	3 (10.34)
	Looking after family or home	1 (3.45)
	Maternity leave	17 (58.62)
Mother’s highest qualification*	University degree or above	24 (82.76)
	A-levels or equivalent	1 (3.45)
	GCSE or equivalent	3 (10.34)
Household Income (GBP)*	20,000–55,000	6 (20.69)
	55,001–80,000	12 (41.38)
	80,001 upwards	10 (34.48)
Marital status*	Married or cohabiting	28 (96.6)

*Missing data = 1 (3.4%)

### Experimental paradigm and procedure

**Fig 1:**
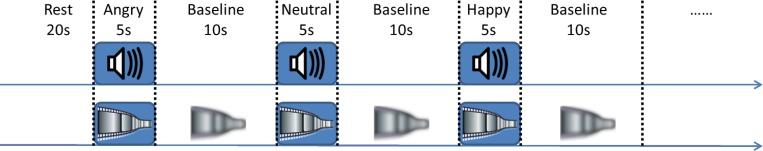
Experiment design. The streamline demonstrates the timeline of the experimental task stimulus presentation and baseline. The task started with a 20-sec rest period, followed by a 5-sec stimulation presented. A 5-sec silent cartoon video was shown during each stimulation presentation trial to attract infant attention and reduce motion artefact. After each stimulation trial, a 10-sec silent blurred, cartoon baseline was presented. The silent cartoon was the same for all the stimulation conditions (angry, neutral and happy).

During the fNIRS experimental procedure (Fig 1), infants sat on their mother’s lap facing a laptop and wearing the NIRS headband. The task started with a 20-sec rest period, followed by a 5-sec trial presented through loudspeakers (SPL = 70 dB). A 5-sec silent cartoon video was shown during each trial to attract infant attention and reduce motion artefact, as consistent with previous research [27]. After each trial, a 10-sec silent blurred cartoon baseline was presented. The task was presented with PsychoPy software [46]. Each condition (angry, happy and neutral) was presented 8 times amounting to a total number of 24 trials. The same emotional expression did not occur consecutively. The testing session lasted 6 minutes and 20 seconds.

### Vocal stimuli

The stimulus material consisted of 15 adult female, non-speech vocalisations of angry, happy and neutral prosody (interjection ‘ah’) from a well-validated battery of vocal emotional expressions [47]. This battery has high internal consistency for each emotion set as well as high levels of specificity (independence between the ratings in the different emotion sets [47]. These stimuli have been validated in previous research in UK children and adults [48] and have been applied in neuroscience studies in typically developing children and children with developmental disorders [35, 49]. Five normalised stimuli, each lasting 1 sec, from the same expression category were selected and combined to form a 5-sec trial. All vocal stimuli were normalised with Praat sound-analysis software [50] to the same duration of 1000 ms and mean intensity of 73 dB.

### fNIRS data acquisition

**Fig 2:**
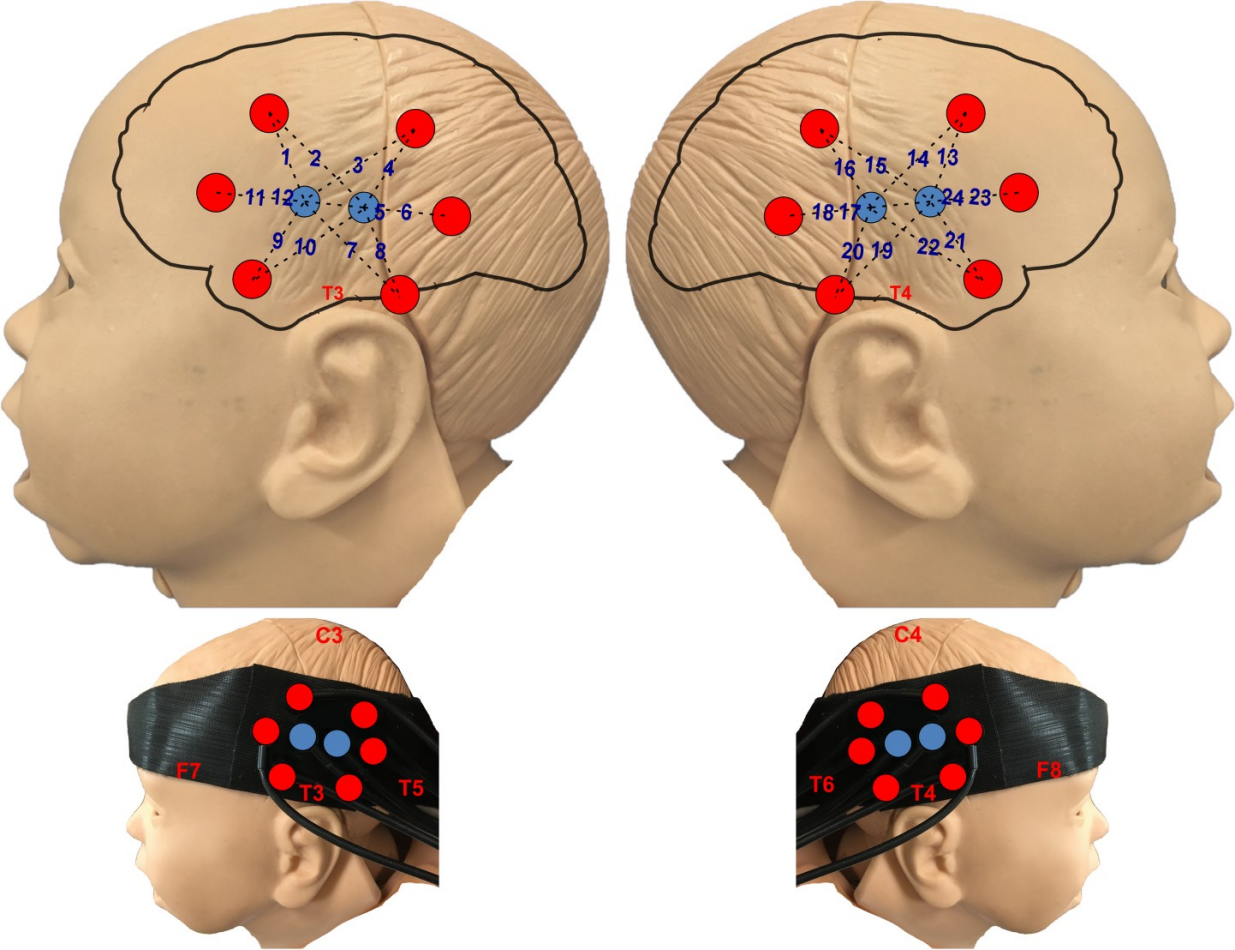
Source-detector distribution. The head model illustrates the source-detector distribution where red dots represent sources (6 in each hemisphere) and blue dots represent detectors (2 in each hemisphere), and are held by Velcro headband. The channel locations with respect to the 10–20 system are marked in red (upper head models). Sources and detectors form 12 recording channels in each hemisphere, which are marked in blue numbers (bottom head models).

During functional cerebral activation, the NIRS setting measures the attenuation of light that corresponds to an increase of Oxy-Haemoglobin concentrations and a decrease of Deoxy-Haemoglobin concentrations in the blood flow [44, 51, 52]. Previous fNIRS studies suggested Oxy-Haemoglobin concentration changes as the most sensitive indicator of changes in cerebral blood flow and has the highest signal-to-noise ratio (see [44, 53]). Although we reported both Oxy- and Deoxy-Haemoglobin concentration changes, we focus our analysis and discussion on the Oxy-Haemoglobin concentration changes. In the present study, infants’ cerebral responses were recorded with a multichannel NIRS data collection system. The system was built by Biomedical Optics Research Laboratory (Dept. of Medical Physics and Bioengineering, University College London) and applied with 780nm and 850nm continuous wavelengths and 10Hz sampling rate [54]. Two detectors and 6 sources formed 12 source-detector pairs in each hemisphere and were distributed at temporal regions, which have been shown to be voice sensitive in previous research in infants [27, 28, 55, 56]; and adults [33, 57, 58]. To achieve the best spatial sensitivity profile for infants [59], the distances between source and detectors were fixed between 1.5 and 2.5 cm. Channels were distributed according to the 10–20 system and attached to a custom-made Velcro headband. The headband was adjusted by calculating the distance between the glabella and the ear, ensuring that T3 and T4 are between the two bottom sources in each hemisphere. The locations of the channels and the channel positions with respect to the 10–20 system are presented in Fig 2. The source-detector geometry was put into the HOMER2 NIRS analysis toolbox (version 2.1, http://homer-fnirs.org/, Huppert et al., 2009[60]) as a matrix. The HOMER2 package then modelled the scattering paths according to the provided parameters.

### fNIRS data analysis

Video-recorded infant behaviour during the task was viewed to code whether the infant attended to the screen without large motion artefacts. Four out of eight trials per condition was set as the minimum criterion for inclusion of each infant dataset.

All the datasets analysed were filtered at 0.01 to 0.5Hz with 3rd order Butterworth filter, to eliminate slow drifts, instrument noise and physiological artefacts, such as heartbeats [27, 61, 62]. The remaining artefacts were identified on a channel by channel basis with the algorithm ‘hmrMotionArtifactByChannel’ implemented in the HOMER2 NIRS toolbox. Within the time interval (tMotion), if the change of the signal amplitude exceeded the threshold (AMPthresh) or the standard deviation changes were greater than a factor (STDEVthresh) multiplied by the original channel standard deviation, the time period (tMask time before and after the motion artefact) was marked as artefact. The time period of motion artefact within the channel was corrected with a cubic spline interpolation algorithm with p set to 0.99 as recommended [62, 63]. Since the algorithm works on a channel by channel basis, the actual standard deviation threshold for the motion artefact varies according to the standard deviation of the original channel; the setting of the STDEVthresh is the multiplication factor rather than a fixed threshold (i.e. in the current study the standard deviation threshold is 20*standard deviation of the channel). This means that the standard deviation threshold varies from channel to channel and subject to subject. All the values were set as follows: tMotion = 5s; tMask = 1s; STDEVthresh = 20; AMPthresh = 5.

After pre-processing, data were converted to Oxy- and Deoxy-Haemoglobin concentration changes (ΔHbO^2^ and ΔHbR) in HOMER2 and averaged across trials in the same emotion condition within each dataset, with the time window of 1 sec before and 15s after the stimulation onset. The averaged time course of each channel was corrected by subtracting the mean of the 1 sec before the stimulation. The analysis focused on ΔHbO^2^ as the most sensitive indicator of changes in cerebral blood flow. Based on earlier work showing that the haemodynamic response reaches the peak around 2 to 4 sec post stimulus [64], we targeted a time window of 2 sec to 9 sec after stimulus onset. Mean amplitudes of cortical haemodynamic responses (ΔHbO^2^ and ΔHbR waveforms) were averaged over the time window of 2 sec to 9 sec after stimulus onset. The averaged haemodynamic responses to the expression conditions (angry, happy and neutral) were evaluated with repeated measures ANOVA and post-hoc pairwise comparisons to find channels sensitive to emotional vocalisations.

We calculated partial eta-squared [65, 66] to estimate the effect sizes for the main effect of emotion as well as for contrasts. Partial eta-squared takes values between 0 and 1. Values of 0.02, 0.13 and 0.26 are indicative of a small, medium, and large effect size, respectively [67].

A false discovery rate (FDR, Benjamini and Hochberg, 1995 [68, 69]) correction was applied to correct multiple comparisons, consistent with other recent infant studies [30, 70]. As the detector array covers a large area of the infant’s brain, we do not expect all detectors to cover brain areas that are responding to our stimulation. Therefore, we only include channels that show a response to the stimulus paradigm. Within identified emotional sensitive channels, pairwise contrasts were corrected with the following steps: (i) A number of p values obtained from post-hoc comparisons (LSD) were arranged with ascending order (from the smallest to the largest) with an order number index, (ii) Adjusted α values were calculated with the equation αadjust = (order index/total number of comparisons)*0.05 and (iii) A comparison was deemed to be significant if the pairwise p value is smaller than the adjusted α value (αadjust) [68, 69]. The significance level is the same as calculated with R code.

### Maternal interaction behaviour

A 6-min mother-infant free play interaction session was video recorded during the same visit following the fNIRS session. Mothers were asked to sit on a floor mat and play with their infant as they would normally do at home optionally using a small set of (supplied) toys. Recording commenced once mother and infant were settled into play. The videos were later coded using the Manchester Assessment of Caregiver-Infant Interaction (MACI [71, 72]), a validated global rating scheme comprising eight 7-point scales suitable for use with normative and at-risk groups [73, 74]. The current study focused on the two caregiver scales, which are normally distributed in a non-clinical population: (1) sensitivity: the degree to which the infant’s behaviour and state are met by prompt, appropriate and attuned responses to meet the infant’s immediate and developmental needs, including an attentive attitude, appropriate engagement and the provision of support and structuring in response to infant behaviour and a lack of behaviour (7-point scale indicates, in order: minimal, occasional, scattered, some, fairly consistent, consistent or high sensitivity). (2) directiveness (reversed in this study from the ‘nondirectiveness’ scale for ease of interpretation): the degree of restrictive or controlling behaviour as characterised by demanding, intrusive, critical and/or other controlling behaviours or comments directed at the infant (7-point scale, indicates in order: highly nondirective, nondirective, mainly nondirective, somewhat nondirective, moderately directive, directive, highly directive). Rating was based on detailed operationalisation of the scale and each rating outlined in the MACI coding manual [71]. A trained and statistically reliable rater (blind to family information and study aims) reviewed the 6-minute videos of mother-infant play at least twice and assigned a 1–7 rating, guided by the MACI coding manual [71] (for further coder training details, see [69], and http://research.bmh.manchester.ac.uk/maci/). Based on the second independent blind coding of 12 (30%) videos, inter-rater agreement was high (intraclass correlation using single measures, absolute agreement definition: sensitivity: r = 0.84; directiveness r = 0.70; both p < 0.001).

## Results

### Emotion effect

Repeated measures ANOVAs with emotion (angry, happy and neutral) as the within-subject factor revealed 3 channels that were sensitive to emotional prosody in ΔHbO^2^: Channel 2 in the left hemisphere (F (2, 56) = 3.38, p = .040, $\eta_{p}^{2}$ = .11); channel 14 in the right hemisphere (F (2, 56) = 3.24, p = .047, $\eta_{p}^{2}$ = .10) and channel 16 in the right hemisphere (F (2, 56) = 4.38, p = .017, $\eta_{p}^{2}$ = .14) (Table 2).

**Table 2:** Infant ΔHbO^2^ change effects in response to vocal emotion: ANOVA on all contrasts.

Channel	Emotion	Mean ± SEM	ANOVA				Pairwise Comparisons			Adjusted α value
			F	p	Partial Eta-squared	Comparison^a^ (A, H and N)	F	p	Partial Eta-squared	α_adjust_
2	Angry	2.82±1.6	3.38	0.040	0.11	A > H	0.56	0.462	0.02	0.044
	Happy	0.97±1.9				A > N	9.76	0.004*	0.26	0.006
	Neutral	-2.68±1.5				H > N	2.86	0.102	0.10	0.033
14	Angry	0.29±1.34	3.24	0.047	0.10	H > A	4.26	0.048	0.13	0.022
	Happy	4.02±1.67				A > N	0.11	0.746	0.004	0.050
	Neutral	-0.33±1.24				H > N	5.62	0.025	0.17	0.017
16	Angry	-1.51±1.74	4.38	0.017	0.14	H > A	8.26	0.008*	0.23	0.011
	Happy	4.49±1.58				N > A	1.10	0.300	0.04	0.039
	Neutral	0.73±1.25				H > N	3.80	0.060	0.12	0.028

* Comparison survived FDR correction (comparisons for which the p values were smaller than the adjusted α value).

^a^ A = Angry, H = Happy, N = Neutral

**Fig 3:**
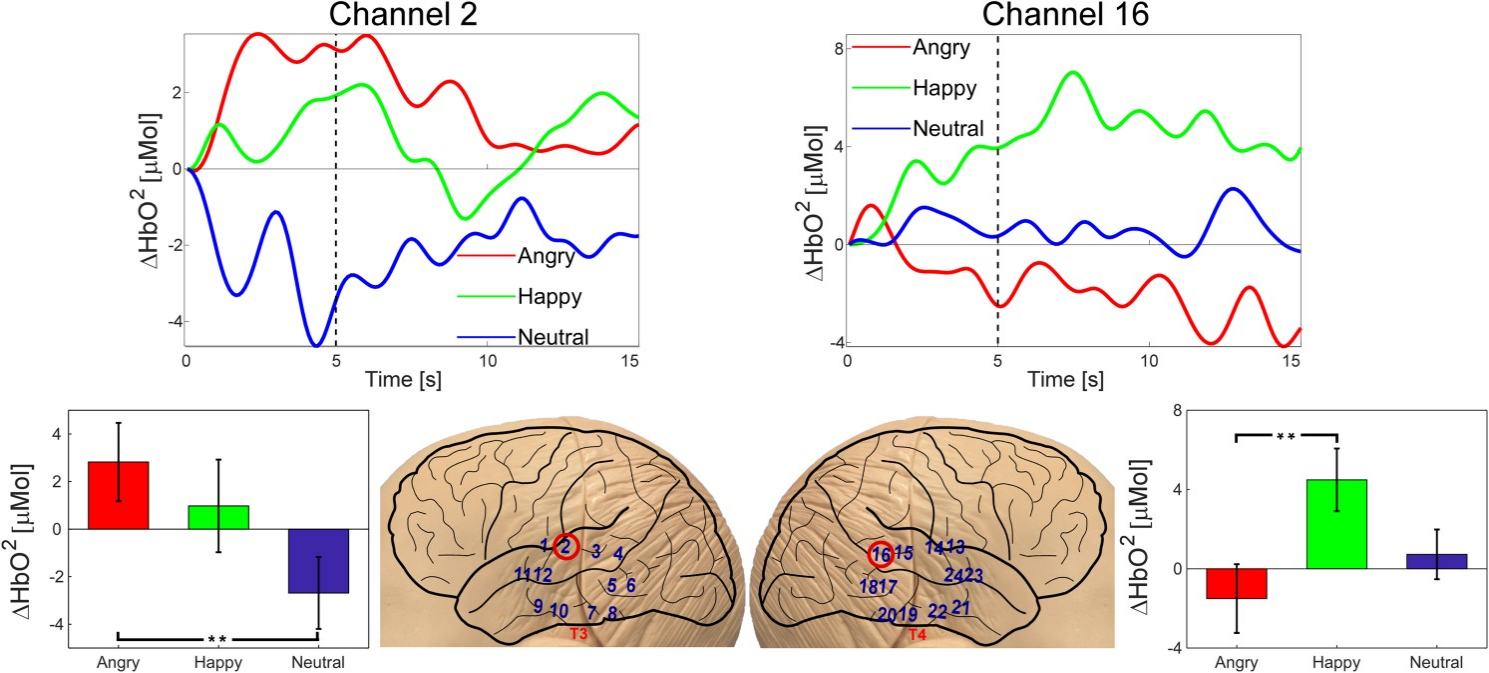
Averaged time courses of ΔHbO^2^ in channel 2 and channel 16. Averaged time courses of ΔHbO^2^ across all datasets in channel 2 and channel 16 per vocal emotion (angry in red, happy in green and neutral in blue) in the time period of 15 sec (5 sec stimulus and 10 sec baseline). The channel location is marked in red in the infant head model. The stimulus offset is marked by the dashed line (at 5 sec). The time (in sec) and change in amplitude (μMol) are in the x and y axis, respectively. The mean and SEM value of ΔHbO^2^ in each channel per vocal emotion is shown in the bar plot. ‘**’ represents the significant (p < 0.01) pairwise comparisons after FDR correction (all the test statistics are presented in Table 2).

Pairwise comparisons showed significant increased ΔHbO^2^ on hearing angry compared to neutral voices (channel 2: F (1, 28) = 9.76, p = .004, $\eta_{p}^{2}$ = .26) and happy compared to angry voices (channel 16: F (1, 28) = 8.26, p = .008, $\eta_{p}^{2}$ = .23) which survived FDR correction (Fig 3). Two further pairwise comparisons did not survive FDR correction (Table 2): happy compared to neutral voices (channel 14: F (1, 28) = 5.62, p = .025, $\eta_{p}^{2}$ = .17) and happy compared to angry voices (Channel 14: F (1, 28) = 4.26, p = .048, $\eta_{p}^{2}$ = .13).

DeoxyHb concentration changes complemented the ΔHbO^2^: 2 channels were sensitive to emotional prosody and survived FDR correction: a significant effect of emotion (left hemisphere: channel 2: F (2, 56) = 4.04, p = .020, $\eta_{p}^{2}$ = .13), particularly in response to angry compared to neutral voice (F (1, 28) = 10.26, p = .003, $\eta_{p}^{2}$ = .27) and a significant effect of emotion in channel 16 in the right hemisphere (F (2, 56) = 3.62, p = .030, $\eta_{p}^{2}$ = .11) in response to happy compared to angry voice (F (1, 28) = 7.45, p = .010, $\eta_{p}^{2}$ = .21).

### Maternal interaction behaviour and infant neural responses

The sample received a broad range of ratings (on a 1–7 scale) for maternal sensitivity (Mean ± SD = 4.17 ± 1.31, range: 2–7) and maternal directiveness (Mean ± SD = 3.93 ± 1.65, range: 1–7). Bivariate correlations tested whether (1) maternal characteristics (current work status, mother’s highest qualification, household Income, and partner cohabitation status) were associated with maternal interaction behaviour ratings; (2) ΔHbO^2^ concentration changes (emotion minus neutral ΔHbO^2^) in the two significant vocal emotion-sensitive areas that survived FDR correction (angry minus neutral ΔHbO^2^ in left hemisphere channel 2; happy minus angry ΔHbO^2^ in right hemisphere channel 16) were associated with maternal interactive behaviour ratings; (3) ΔHbO^2^ concentration changes were associated with maternal characteristics.

**Fig 4:**
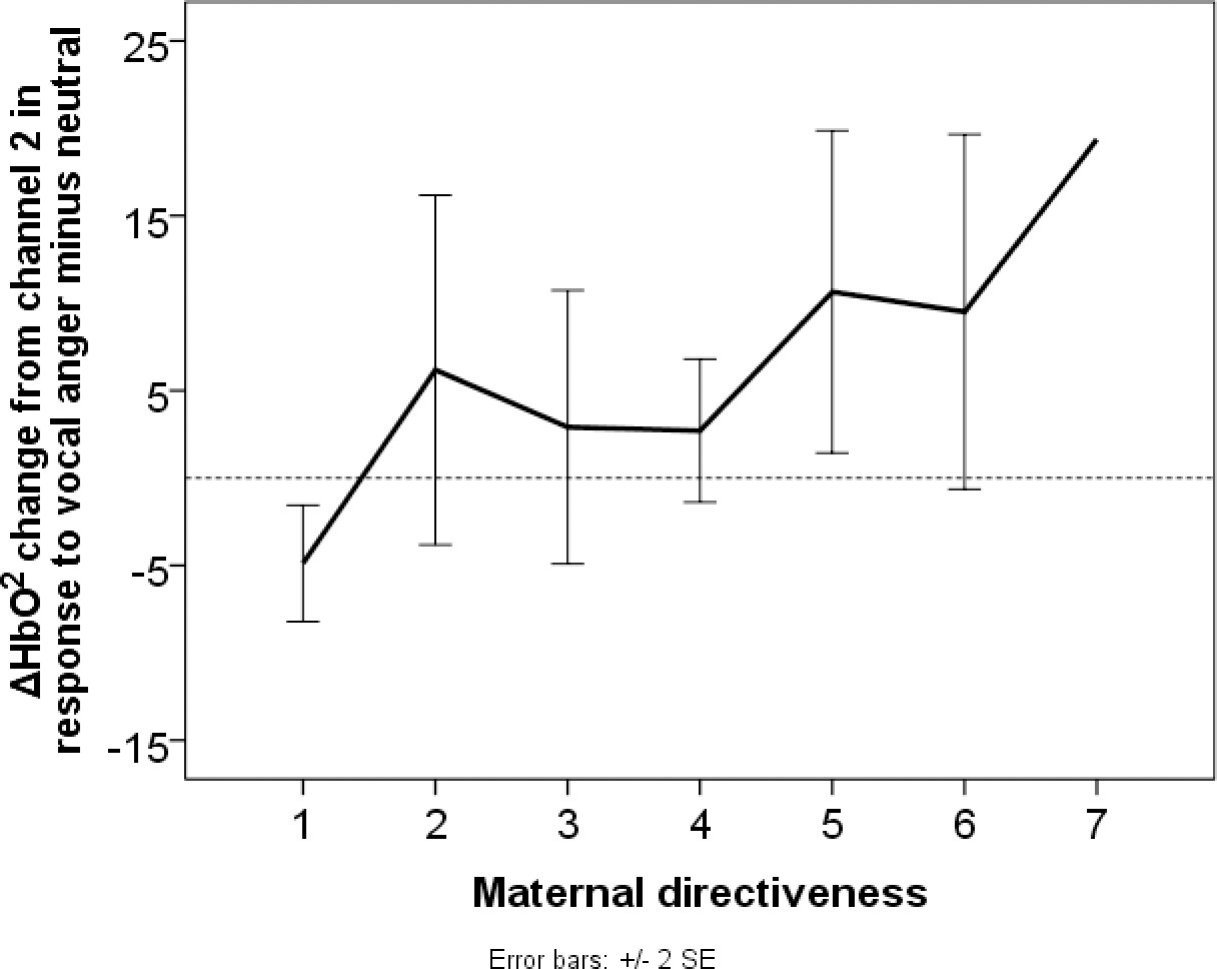
Association between neural responses to angry minus neutral prosody and maternal directiveness. Infant neural response to angry minus neutral vocalisations (y axis) increases linearly with independent ratings of how directive mothers were towards their infant during play interaction (7 = highly directive; x axis). The black hard line represents the mean HbO^2^ change for each rating on the maternal directiveness scale.

Only one significant correlation was found between maternal interaction behaviour ratings and maternal characteristics: maternal sensitivity was positively correlated with maternal highest qualification (r = 0.41, p = 0.028). Although ΔHbO^2^ in neither region was associated with maternal sensitive responsiveness, increased activation to angry minus neutral prosody was negatively correlated with maternal directiveness: r = 0.41, p = 0.029 (Fig 4). ΔHbO^2^was not associated with any of maternal characteristics.

## Discussion

This is the first study of infant neural processing of emotional non-speech prosody to demonstrate the heightened recruitment of bilateral temporal cortices at 6 months in response to vocal emotion. It suggests that at least part of the temporo-frontal network recruited in adult vocal emotion processing [19–22] is already functioning by 6 months of age. More broadly, our findings are consistent with previous behavioural and neuroimaging findings that 6-month-old infants can distinguish emotional from neutral sounds and between basic emotions (or emotional valence), irrespective of speech [27, 29, 30, 36]. We also offer preliminary evidence of statistical link between negative (angry) vocal discrimination in the temporal region and early social or caregiving experience. Specifically, hearing angry vocalisations evoked stronger responses in the left anterior superior temporal cortex (STC) compared to neutral prosody and infants with stronger activation in this vocal anger-sensitive region experienced more directive interactions from their mother. Happy prosody evoked increased activation in the right posterior (and possibly anterior) STC compared to angry prosody. However, the strength of this response in the right temporal cortex was not associated with our measures of maternal social interaction.

Our main findings are consistent with previous infant brain studies that implicate the temporal cortices [27, 36, 75], broadly supporting the temporo-frontal network. Angry and happy prosody evoked left and right STC activations that seem to show distinct cortical activation to emotional stimuli. Rather than a laterality effect, this activation difference is likely to be an artefact of strictly correcting multiple comparisons; thus, we would suggest that the uncorrected results may reflect a broader bilateral STC activation in response to emotional vocalisations generally. Evidence from adult studies suggests that STC is sensitive to emotional vocalisations and the STC activation is not associated with emotional valence [21, 25, 26]. While previous studies implicate a frontal asymmetry in infants’ responses to emotional stimuli [76, 77], evidence to date on the hemispheric lateralisation of effects in response to emotional sounds, especially in the temporal region, is heterogeneous in infant studies. Infant ERP studies found bilateral frontal, temporal, and central activations in response to emotional speech and emotional non-speech sounds [36, 78], and fNIRS studies reported right superior temporal and right inferior frontal activations to emotional speech [27, 37]. Neuroimaging evidence in adult studies also support both right hemisphere and bilateral involvement in vocal emotion processing [20–26]. Given the range of previous findings and the lack of infant frontal measurement in the present study, we did not hypothesise any laterality effect. The lack of clear lateralisation effect in our study may reflect the relative immaturity of the temporal cortices at 6 months of age when infant neural sensitivity to vocal emotions may not yet be stable or specialised. The superior temporal cortices are known as part of the social brain that undergo an experience-dependent “fine tuning” process into specialised functions [27]. Furthermore, the current study focused on non-speech prosody, reflecting how mothers commonly express themselves to preverbal infants, while previous infant studies measured neural responses to emotion in speech. Emotional information carried in speech may be confounded by the high variation in how much semantic understanding 6-month-old infants have of the speech content (i.e. receptive language, [79, 80]).

We report that hearing angry vocalisations evoked a response localised to the left anterior STC, which may reflect a general negativity attentional bias that is seen in adults [34, 48, 81–83]. Neural sensitivity to angry compared to neutral voice has also been reported in other infant studies [27, 36, 75], raising the question of whether a prioritised neural response to threatening vocal information may be innate, consistent with evolutionary explanations [2]. An imaging study of vocal emotion processing in sleeping neonates suggests that an automatic perception of threat-related emotional voices may be active from birth [81], and our findings may reflect a conscious attentional process present at 6 months of age, as reflected in the recruitment of the left STC. Contrary to expectations, neural responsiveness to happy compared to neutral prosody in the anterior STC (channel 14) did not survive FDR correction, possibly suggesting that this localised happy-specific sensitivity is not (yet) stable developmentally or may only be present in a subgroup. A larger sample may provide us with the statistical power to observe greater neural responses to happy vocalisations compared to neutral. An alternative interpretation may be that channel 14 is responsive to emotional valence from negative (angry) to positive (happy) and therefore found happy vocalisations a stronger contrast with angry than with neutral vocalisations. However, the right posterior STC activation to happy compared with angry prosody is consistent with right lateralised effects found in other infant and adult studies on vocal emotion [20–22, 27].

With respect to the second objective, we found that infant neural sensitivity to prosodic anger was associated with degree of maternal directiveness. Maternal directiveness typically involves vocal and behavioural demands, intrusions and/or critical utterances, which requires the infant’s behavioural responses (such as an adjustment of the attention, and/or a change of the current behaviour). A mother may use directive behaviours to teach, guide, or direct the infant to behave and/or play in a socially acceptable way. Our findings require replication in a larger sample but provide preliminary evidence that may suggest that early social experience in the form of directive caregiver interactions, or stress that may result from such interactions, may promote cortical specialisation in vocal anger perception. Although not all directiveness carries vocal negativity, being the recipient of high caregiver directiveness is likely to involve appraising negative emotion more often as a guide to acceptable behaviour, and, therefore may plausibly heighten the STC processing of negative prosody. Since maternal and infant anger were not directly measured in this study, whether more directive caregivers actually used more anger vocal expressions and/or whether their infants experienced more anger (or irritation) as a result of their social interactions is unknown. In addition, few mothers in this study were rated as particularly high or low in directiveness, and, therefore, the effects may be stronger in a sample recruited specifically to test out associations with maternal behaviour.

On the other hand, we found no association between maternal sensitivity behaviour and neural response to emotional prosody in our 6-month-old infants, suggesting that infant neural processing of vocal emotions does not vary according to infant experience of maternal sensitivity, at least in the typically developing infants of healthy mothers. While maternal directiveness conceptually overlaps with emotional negativity, high maternal sensitivity does not always entail emotional positivity, but rather affect is attuned (i.e. well-modulated to infant affect) and generally well matched–for example, if the infant is fretful, then warm but not affectively positive interactions would constitute a sensitive response. Statistically, in the current sample, the distribution of ratings was slightly narrower for maternal sensitivity (ratings were mostly centred at the medium), which may have also reduced the likelihood of finding a statistical association. It is possible that significant effects may only be seen in a clinical or at-risk group which may have more variation in maternal sensitivity ratings.

Several methodological considerations must be taken into account in the interpretation of our findings. First, the present study included a relatively modest overall sample size. Although comparable with other similar imaging studies of infants, it precludes analysis of gender effects to take account of known early gender differences in vocal emotion processing [84, 85]. Secondly, the study focused on effects in the temporal cortical regions and did not investigate the involvement of other (e.g. frontal) regions implicated in vocal processing [86]. Thirdly, since we used only angry and happy emotional stimuli, the anger-related effects reported may result from emotional negativity in general, rather than being anger-specific. Fourthly, distinctive neural patterns to emotional categories do not necessarily suggest a conceptual understanding of emotions by infants, although experimental findings indicate that discrete emotions are at least paired with different kinds of infant responses or preferences [12–15], suggesting a level of evaluative appraisal rather than solely an acoustic analysis of pitch characteristics by the infant. A combined fNIRS and experimental approach (such as eye-tracking) would provide supportive infant attentional data, providing further understanding of whether neural responses to vocal emotions correspond to infant behaviours. Finally, we did not test infants’ hearing ability directly but relied on maternal report.

In conclusion, we report novel evidence that prosodic anger elicited STC activation in 6-month-old infants, has also been implicated in adult vocal emotion perception. This is consistent with an important function for vocal emotion perception in the first year of life in guiding communicative and relational development. Furthermore, we report the first preliminary evidence of an association between infant brain responsivity to vocal anger and maternal directiveness in a healthy sample. Replications in larger samples of infants, and in high risk groups (e.g. mothers with mental illness), as well as further investigation of this association may help us understand better the role of early experience on vocal perception as a building block for communicative and socioemotional development. Future studies should also consider broader and more specific environmental influences on infant vocal emotion processing by linking the fNIRS data with infant exposure to maternal and non-maternal positive and negative affect within naturalistic vocalisations and speech, for example, by collecting day-long samples of audio recordings at home. The current paradigm may be developed to evaluate the effectiveness of parenting interventions on neural sensitivity to vocal emotion in healthy and at-risk groups early in infancy. Such interventions may be designed to target caregiver directiveness to help unravel the directionality of effects. Future research employing longitudinal designs could also be useful to follow the developmental trajectories of neural sensitivity to emotional vocalisations in typical development to assess its potential as a biomarker of atypical neurodevelopment in at-risk children [87].

## Supporting information

S1 File Dataset. (ZIP)
